# Dynamic contrast-enhanced CT and clinical features of sarcomatoid hepatocellular carcinoma

**DOI:** 10.1007/s00261-023-03983-1

**Published:** 2023-07-10

**Authors:** Guangming He, Weiqing Huang, Zhimei Zhou, Hui Wu, Qin Tian, Lilian Tan, Xi Li

**Affiliations:** 1 Department of Radiology, The Second Affiliated Hospital of Guangzhou Medical University, Guangzhou Medical University, No.250, Changgang East Road, Haizhu District, Guangzhou, 510399 Guangdong China; 2 Guangzhou Medical University, Guangzhou, 511495 Guangdong China; 3 Department of Pathology, The Second Affiliated Hospital of Guangzhou Medical University, Guangzhou Medical University, Guangzhou, 510399 Guangdong China

**Keywords:** Sarcomatoid hepatocellular carcinoma, Hepatocellular carcinoma, Computed tomography, Pathology

## Abstract

**Purpose:**

To investigate the dynamic contrast-enhanced computed tomography (CECT) features and clinical characteristics of sarcomatoid hepatocellular carcinoma (S-HCC).

**Methods:**

We retrospectively reviewed the CECT data and clinical findings of 13 patients (11 male and 2 female, with an average age of 58.6 ± 11.2 years) with pathologically proven S-HCC, including 9 patients with surgical resection and 4 patients with biopsy examination. All patients underwent CECT scans. Two radiologists reviewed and evaluated general features, CECT features and extratumoral features of each lesions based on a consensus.

**Results:**

Among the thirteen tumors, a mean size of 66.7 mm was observed, ranging in diameter from 30 to 146 mm. Seven of thirteen patients had hepatitis B virus (HBV) infection and an elevation of alpha-fetoprotein (AFP) level. Most of cases located in the right lobe of liver (84.6%, 11/13). Nine of thirteen tumors showed lobulated or wavy contours and infiltrative morphology, while eight tumors presented with unclear margin. The tumor textures were mainly heterogeneous for ischemia or necrosis, with solid components dominantly in all cases. Eight of thirteen tumors exhibited “slow-in and and slow-out” dynamic enhancement pattern in CECT, with a enhancement peak in the portal venous phase. Portal vein or hepatic thrombus, adjacent organs invasion and lymph node metastasis were observed in two patients, respectively. Four of thirteen lesions occurred intrahepatic metastasis and hepatic surface retraction respectively.

**Conclusion:**

S-HCC gengerally seen in elderly male with HBV infection and elevated AFP level. The CT manifestations including: large diameter, frequently hepatic right lobe involvement, lobular or wavy contours, ill-defined margins, infiltrative morphology, obvious heterogeneity and dynamic enhancement pattern of “slow-in and and slow-out” , contributed to the diagnosis of S-HCC. These tumors usually occurred hepatic surface retraction and intrahepatic metastasis.

## Introduction

Primary hepatic sarcomatoid carcinoma (PHSC) is a rare malignant tumor, defined as a tumor composed of both carcinomatous (either hepatocellular or cholangiocellular) and sarcomatous components, which accounts for only 0.2% of the primary malignant liver tumors [1]. PSHC containing hepatocellular or cholangiocellular components were named as sarcomatoid hepatocellular carcinoma (S-HCC) or sarcomatoid intrahepatic cholangiocarcinoma (S-ICC) respectively [1]. Previous studies have revealed that the prevalence of S-HCC and S-ICC is 0.27–9.4% of HCC and 4.5% of ICC [2–6].

It was reported that S-HCC has an extremely poor prognosis, a high incidence of metastases and early recurrences compared to conventional HCC or ICC [2, 7, 8]. Moreover, repeated non-surgical therapy such as radiofrequency ablation and transcatheter arterial chemoembolization (TACE) resulting in the necrosis and degeneration of hepatocytes, may lead to S-HCC [4, 9, 10]. Currently, apart from radical resection, there has been no effective treatment or prognosis prediction model established for this rare tumor, with scarce data for decision-making and translational studies. Thus, identifying S-HCC is important for proper patient management and treatment planning. However, as the literature was restricted to either small case series or case reports [11–13], there was an unclear concept of CT imaging features of S-HCC, leading to a dilemma for clinical diagnosis of S-HCC preoperatively.

In this study, we aim to further characterize these tumors by reporting the CT manifestations and clinical findings of a series of 13 S-HCC patients, which contributes to an improved understanding and diagnosis of these rare tumors.

## Materials and methods

### Patients

This study was approved by the Institutional Review Board of our hospital, and the informed consent was waived as it’s retrospective nature. A total of 18 patients who underwent dynamic contrast-enhanced CT (CECT) examination and were pathologically confirmed S-HCC by surgical resection or biopsy from January 2009 to August 2022 at our hospital were included. We reviewed the medical records of all patients, collecting the following clinical and pathological data: gender, age, complaint, the preoperative laboratory findings including serum alpha-fetoprotein (AFP) level, hepatitis B or C immunology, serum carbohydrate antigen 19-9 (CA19-9) and carcinoembryonic Antigen (CEA) level, and all preoperative imaging data of CECT. The inclusion criteria were: (1) patients underwent abdominal unenhanced and CECT scans before pathological examination. (2) patients without any treatment or invasive operation before CT examination. (3) patients were confirmed S-HCC pathologically by surgical resection or biopsy. The exclusion criteria were: (1) hard to assess special features due to CT images artifacts (*n*=2); (2) time interval between CECT scan and pathological examination of more than two weeks (*n*=1); (3) incomplete laboratory and pathological results (*n*=2). Finally, a total of 13 S-HCC patients (9 patients with surgical resection) or (4 patients with biopsy) were enrolled in this study.

### Imaging acquisition and analysis

Preoperative CECT images were obtained on multiple scanners: 320-slice spiral Aquilion ONE CT scanner and 256-slice GE revolution CT scanner. The scanners were set as the following parameters: tube voltage 120 kVp, 200 mAs, and a reconstruction slice thickness of 5 mm. After collecting the unenhanced images, each patient was injected with intravenous nonionic iodinated contrast agent (iodipamide, 370 mg I/mL, Bracco) via the antecubital vein by mechanical power injectors based on their weight (2.0 mL/kg body weight, with a maximal dose of 180 ml), followed by a 20-mL saline flush. Finally, three-phase CECT scans images were obtained, including arterial phase (AP), 25–40 s; portal venous phase (PVP), 50–60 s; and equilibrium phase (EP), 120–250 s.

All images were reviewed respectively by two boarded radiologists with 5 and 15 years of experience in hepatic imaging, who were blinded to all information of patients. The following CT features were evaluated and recorded in each tumor when they were in consensus: (1) tumor general features, including tumor amount (multiple or single), specific location in liver, maximal diameter, contour (lobulated/wavy or round/oval), margin (clear or indistinct), morphology (massive expanding or infiltrative), liver cirrhosis, portal vein or hepatic vein thrombus, blood products or fat in mass. (2) CECT features, including major components (solid or cystic), tumor texture (heterogeneous or homogeneous); intratumoral arteries (observed in AP); capsule (a smooth, uniform, sharp border at CT that encloses most or all of a tumor, classified as absent, complete or partial), and the following quantitative CECT parameters: the attenuation of tumors in each CT phase; the enhancement degree of arterial phase (EDA), portal venous phase (EDP), and equilibrium phase (EDE), and dynamic enhancement pattern; (3) Extratumoral features, including invasion of adjacent organs, lymph node metastasis, intrahepatic metastasis, hepatic surface retraction, biliary dilatation (the biliary diameter > 2 mm) and ascites.

The two radiologists measured the CT values of the solid part of each lesion (excluding fat, blood products, calcification and necrotic areas) three times in each phase, obtaining average attenuation of each tumor. Tumors were considered homogeneous if they had a same attenuation area of over 90%. The enhancement degree were defined by the CT attenuation of EDA, EDP and EDE subtracted the CT attenuation of unenhanced phase in each tumor, respectively (enhancement positive: > 10 Hu, mild enhancement: 10 Hu-30 Hu; moderate enhancement: 31 Hu-50 Hu; significant enhancement: > 50 Hu). Dynamic enhancement pattern contained the following three types: (1) “wash-in and wash-out”, tumor showed moderate or significant enhancement in AP and decreased enhancement rapidly in PVP or DP, with a peak of enhancement in AP; (2) “ wash-in and slow-out”, tumor were moderate or significant enhancement in AP or PVP, and decreased enhancement slightly in DP, with a peak of enhancement in AP or PVP; (3) “slow-in and slow-out”, tumor exhibited mild enhancement in AP and moderate or significant enhancement in PVP, but decreased enhancement slightly in DP with a peak of enhancement in PVP. The above CT features were evaluated and confirmed by two senior radiologists who were in consensus.

### Pathology analysis

Pathology for PHSC was based on the histopathological examination of surgical specimens for 8 patients and the percutaneous biopsy for 5 patients, which containing histopathologic findings and immunohistochemical results. For each tumor, the following histopathological factors were assessed: gross type; histological type; necrosis or hemorrhage; vascular invasion; intrahepatic metastasis; presence of lymph node metastasis and ki-67 score. Upon microscopic examination, the tumors mostly exhibited spindle (sarcomatous component), pleomorphic or bizarre giant cells, which were positive for vimentin and keratin, negative for E-cadherin, accompanied HCC with moderate to poor differentiation [1, 4, 14]. Two senior pathologists analysed and confirmed all specimens in consensus.

## Result

### Baseline clinical and histological characteristics

The detailed clinical findings and pathological data are summarized in Table 1. A total of 13 patients confirmed as S-HCC by histopathological examination were enrolled in this study, including 11 males and 2 females, with an average age of 58.6 ± 11.2 years old (rage 39–81 years old). The most common complaints were abdominal discomfort (38.5%, 5/13). The majority of patients were detected incidentally during their routine physical examinations without any symptoms (46.2%, 6/13). The elevation of AFP was found in 7 patients (53.8%, 7/13) and 2 patients were found with an increasing of CA19-9 and CEA, respectively. More than half of patients were diagnosed with hepatitis B virus (HBV) infection (53.8%, 7/13) and 2 patients had liver cirrhosis. The Ki-67 level of all lesions were more than 30%, and the highest one reached 90%, with an average of 62.7% ± 22.2%.

**Table 1:** Clinical characterisrics of S-HCC patients

Patient Number	1	2	3	4	5	6	7	8	9	10	11	12	13
Age	66	59	62	45	75	56	58	59	54	58	50	81	39
Gender	M	M	M	M	M	F	M	M	M	M	M	F	M
Amount	Single	Multiple	Single	Single	Multiple	Multiple	Multiple	Single	Single	Multiple	Single	Single	Multiple
MD (mm)	58	30	75	110	41	52	146	53	111	39	40	70	43
Location	S4、S8	S7	S6、S7	S6、S7	S7、S8	S4-8	S5、S6	S4	S4-8	S5、S6	S2、S3	S5	S6
AFP (ug/L)	29.11	863.48	637.31	1.91	3.09	1.84	0.99	4.26	35.92	2005.00	12108.00	6.54	868.3
CA199 (U/ml)	17.13	10.3	16.76	2.91	5.86	1.84	2.03	10.76	57.03	14.61	28.42	12.03	8.39
CEA (ug/L)	5.26	1.62	3.99	0.7	1.41	0.98	2.67	2.32	6.59	2.43	4.78	2.58	3.06
Virus infection	HBV	HBV	Absent	Absent	Absent	Absent	HBV	HBV	HBV	HBV	Absent	Absent	HBV
Liver cirrhosis	Absent	Absent	Absent	Absent	Absent	Absent	Absent	Absent	Present	Absent	Absent	Absent	Present
Histologic diagnosis	S-HCC	S-HCC	S-HCC	S-HCC	S-HCC	S-HCC	S-HCC	S-HCC	S-HCC	S-HCC	S-HCC	S-HCC	S-HCC
Confirm	Resection	Resection	Resection	Biopsy	Biopsy	Biopsy	Resection	Resection	Biopsy	Resection	Resection	Resection	Resection
Ki-67 (%)	70	30	90	40	35	80	60	60	80	90	90	50	40

*M* male, *F* female, *MD* Maximal diamet, *AFP* alpha-fetoprote, *CA19*-9 carbohydrate antigen 19, C*EA* carcinoembryonic antig, *HBV* hepatitis B virus, *S-HCC* sarcomatoid hepatocellular carcinoma

#### CT general features

**Fig. 1:**
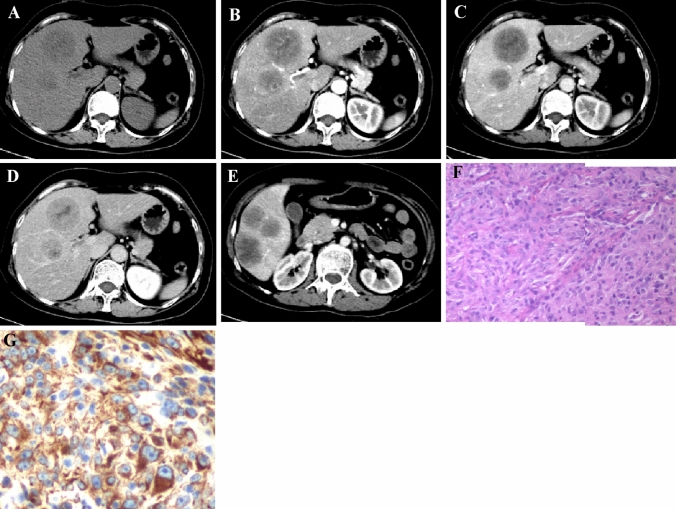
A 56-year-old female with Sarcomatoid hepatocellular carcinoma were found as right upper abdominal pain for one week without the increase of AFP, CA19-9, CEA and HBV infection. The largest tumor in the right lobe of the liver was round, massive expanding with clear margin (**A**). Enhancing in the pattern of “slow-in and slow-out”, the tumor was slightly and persistently enhancing in the arterial phase(**B**), portal venous phase (**C**) and equilibrium phase (**D**), with central necrosis non-enhancement. Intrahepatic metastasis can be seen in the liver (**E**). Hematoxylin and eosin (**H** and **E**) stain showing tumor was composed of spindle and epithelioid cells that were intensively distributed, and clumped chromatin and nucleolus can be seen (**F**, ×200). This tumor had positive Vimentin (**G**), with Ki-67 level reaching 80% (picture was not shown)

**Fig. 2:**
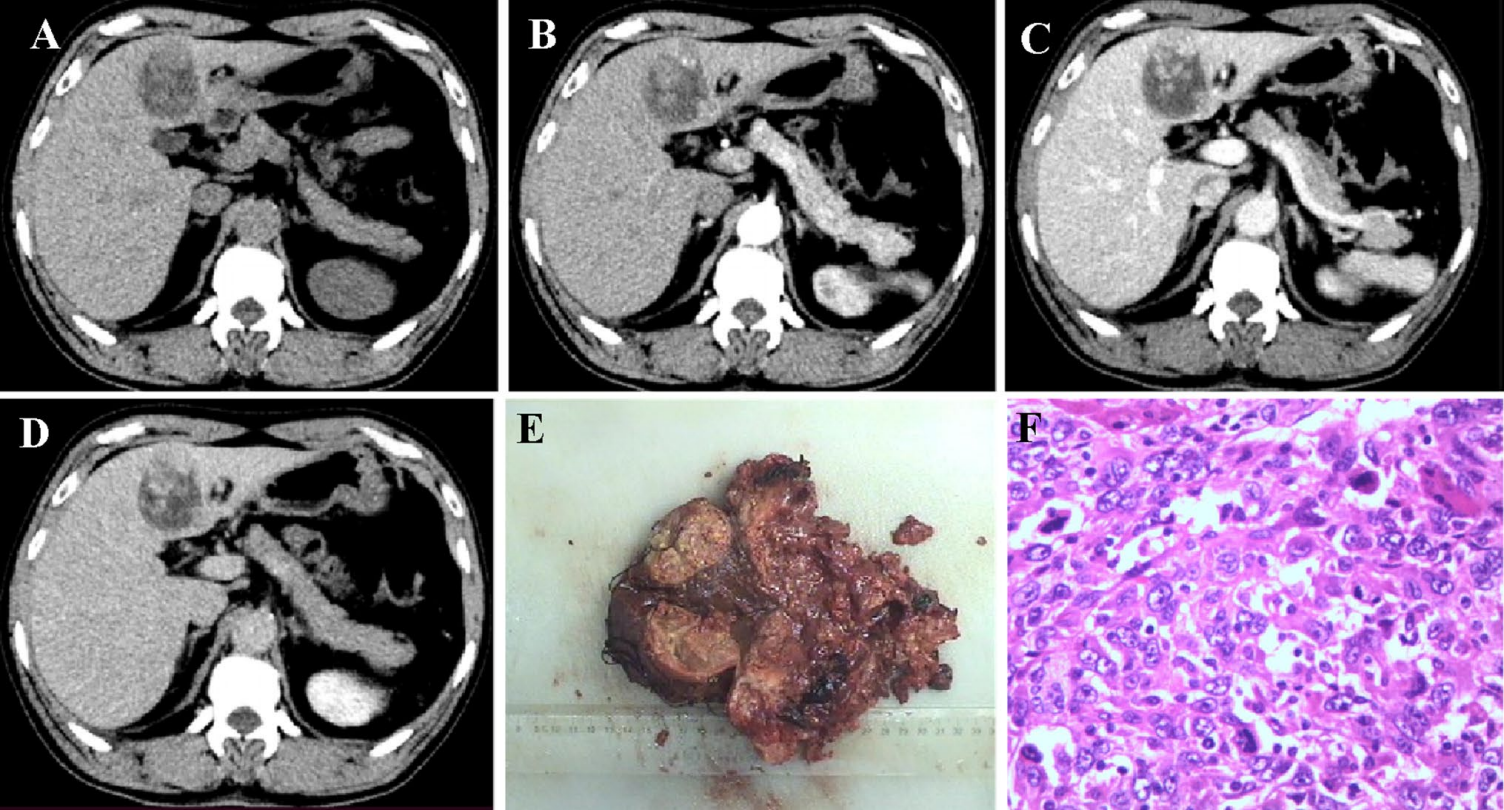
A 53mm Sarcomatoid hepatocellular carcinoma in a 59-year-old man with HBV infection and absence of evaluation of AFP, CA19-9 and CEA. The round and heterogeneous tumor was located in the segment 4 of liver, with well-defined margin and massive expanding morphology (**A–D**). CECT showed the lesion exhibited “slow-in and slow-out” dynamic enhancement pattern (**B**–**D**), which was partial enhancing mildly on the arterial phase (**B**), progressive enhancing on the portal venous phase (**C)** and equilibrium phase (**D, **while arrow) in a larger region. The gross specimen showed a multinodular mass, corresponding to the enhancement region in CECT (**E)**. Some spindle cells can be seen in H &E stain (**F**, ×400). The Ki-67 level of this tumor was 60%, with Vimentin (+)

**Fig. 3:**
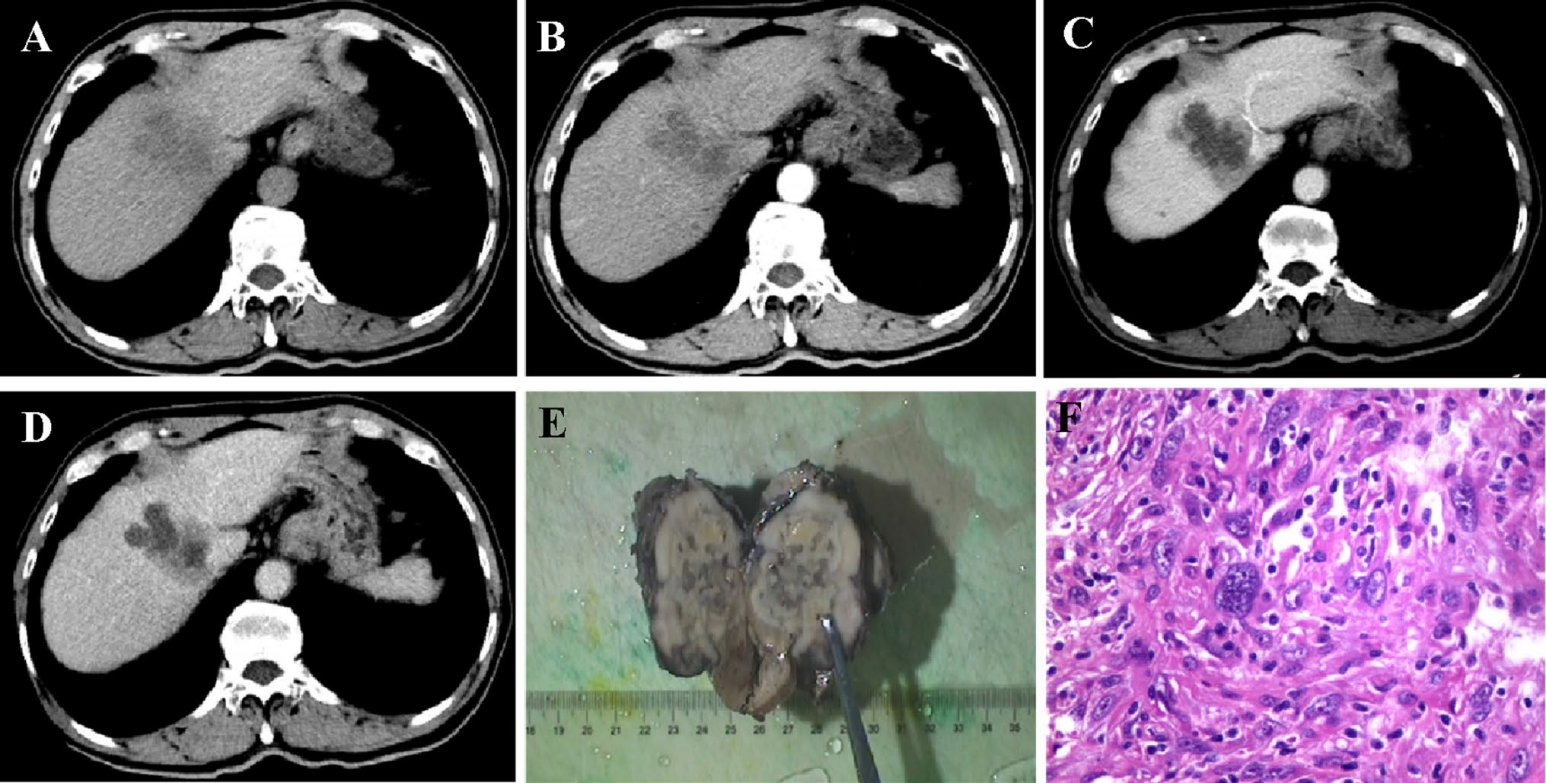
Sarcomatoid hepatocellular carcinoma in a 66 year-old man who was asymptomatic with mild elevation of AFP (29.11 ug/L) and HBV infection. Locating in the right lobe of live, the lobulated and heterogeneous mass (**A**) in a size of 58mm × 48mm, with an infiltrative morphology demonstrated “slow-in and slow out” dynamic enhancement pattern (**B**–**D**), which presented with peripheral enhancing slowly on the arterial phase (**B**) , followed by centrally progressive enhancing on the portal venous phase (**C)** and equilibrium phase (**D**, while arrow). The bisected specimen showed a hard solid and partially necrotic white to grayish mass (**E**). H & E stain showed the neoplastic cells composed of epithelial cells and a prominent component of spindle cells, with scattering bizarre giant cells (poorly differentiated cell) (**F**, ×400). The Ki-67 level of this tumor was 70%, with Vimentin (+)

The general imaging features of S-HCC are summarized in Table 1 and Table 2. Tumor ranged in maximal diameter from 30 to 146 mm with a mean value of 66.7mm. Most of cases located in two or more hepatic segments, with an obvious dominance (84.6%, 11/13) in the right lobe, as shown in Figures 1, 2, 3. There were 6 cases presenting with multiple lesions and 7 cases presenting with single mass. Regarding to tumor contour, 9 tumors exhibited lobulated or wavy contours while 4 tumors were round or oval shaped. 8 cases showed unclear margin and 5 cases were well-defined. The morphology of 9 tumors were infiltrative shape and 4 tumors were massive expanding pattern. Portal vein or hepatic vein thrombus on CECT were observed in 2 cases. There was 1 case having peritumoral hemorrhage, and no fat density were found in all lesions.

**Table 2:** CT General features of S-HCC patients

CT general features	Case number
Contour	
Round/oval	4
Lobulated	9
Margin	
Clear	5
Indistinct	8
Morphology	
Massive expanding	4
Infiltrative	9
Portal vein or hepatic vein thrombus	
Present	2
Absent	11
Fat in mass	
Present	0
Absent	13
Blood products	
Present	1
Absent	12

#### CECT features

**Fig. 4:**
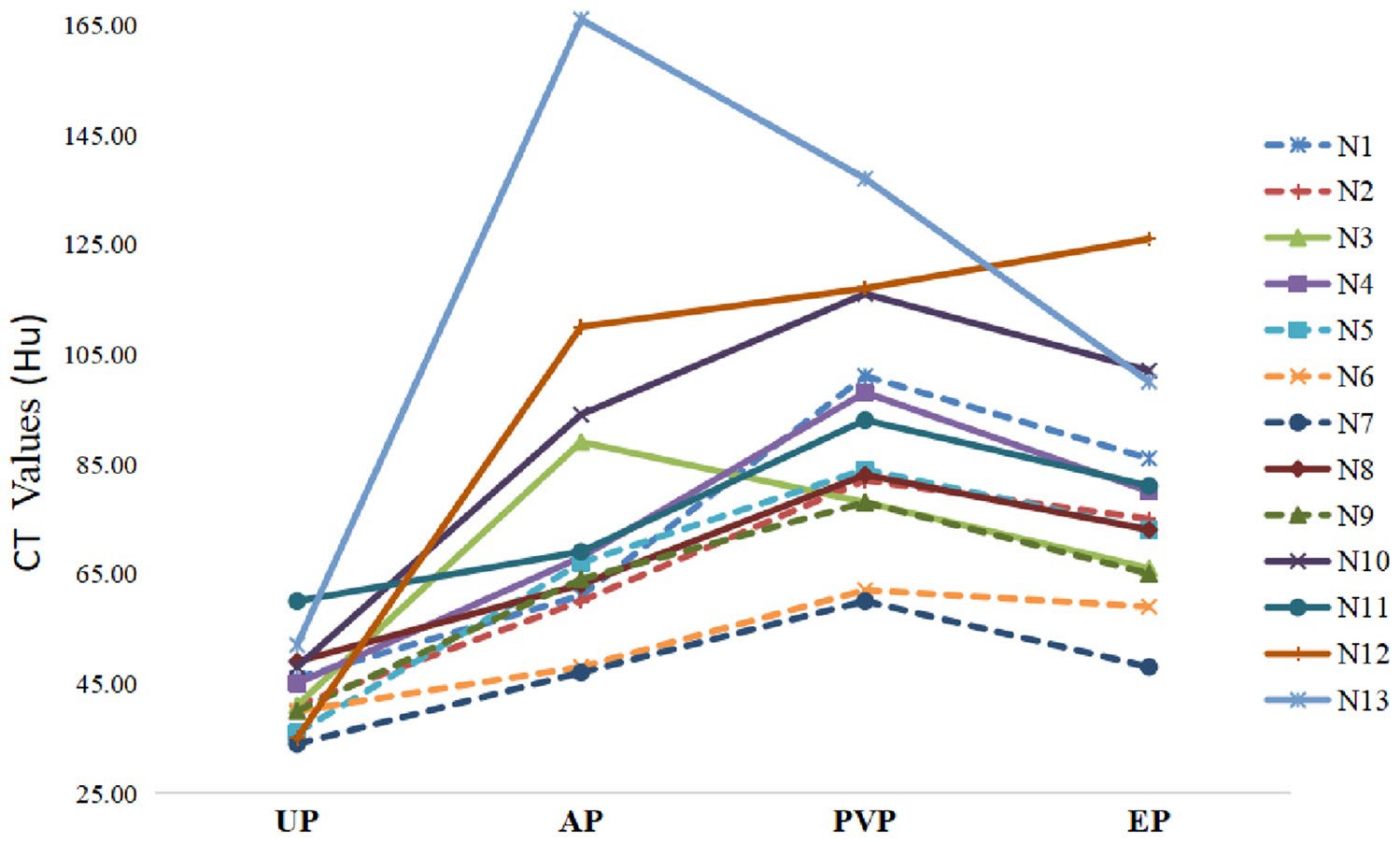
The attenuation of tumor major components in each CECT phase. *UP* unenhanced phase, *AP* arterial phase, *PVP* portal venous phase, *EP* equilibrium phase, *N* number

**Fig. 5:**
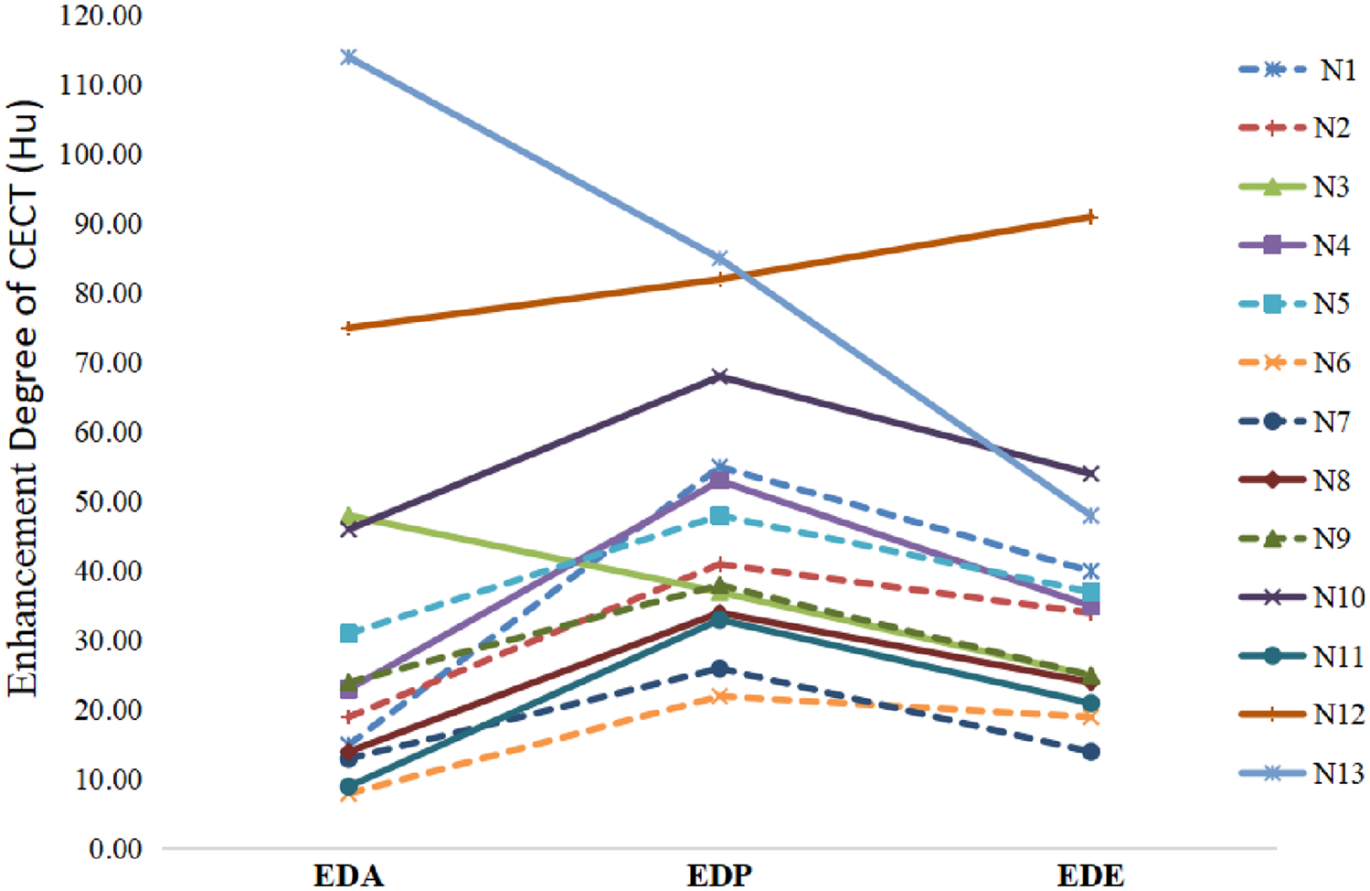
The enhancement degree of arterial phase (EDA), portal venous phase (EDP), and equilibrium phase (EDE). *N* number

The tumor textures were mainly heterogeneous (53.8%, 7/13) for ischemia or necrosis within the tumor center, with solid components dominantly in all cases, as shown in Table 3. The quantitative CECT parameters including the CT values, enhancement degree of the four phases of CECT and the dynamic enhancement pattern of all lesions were demonstrated in Table 4, Figure 4 and Figure 5. The enhancing peak of 8 lesions (61.5%, 8/13) were in the PVP. Most lesions showed “slow-in and and slow-out” (61.5%, 8/13) enhancement pattern, which presented with peripheral enhancing slowly on the AP, progressive or persistent enhancing towards the tumor center in the PVP and mild descending enhancement in the EP (Figures 1, 2, 3). While 3 cases showed “wash-in and slow-out”, and 2 cases showed “wash-in and wash-out” enhancement pattern. Intratumoral arteries were seen in 4 cases during AP. Regarding to capsule, there were 5 lesions with capsule, 4 lesions among which with partial capsule, and 8 lesions had no capsule.

**Table 3:** CECT features of S-HCC patients

CECT features	Case number
Major components	
Solid	13
Cystic	0
Tumor texture	
Homogeneous	6
Heterogeneous	7
Intratumoral arteries	
Present	4
Absent	9
Capsule	
Absent	8
Partial	4
Complete	1

**Table 4:** Extratumoral features of S-HCC patients

Extratumoral features	Case number
Invasion of adjacent organs	
Present	2
Absent	11
Lymph node metastasis	
Present	2
Absent	11
Intrahepatic metastasis	
Present	4
Absent	9
Hepatic surface retraction	
Present	4
Absent	9
Biliary dilatation	
Present	0
Absent	13
Ascites	
Present	3
Absent	10

#### Extratumoral features

On preoperative CECT imaging of all patients, 2 tumors were accompanied by adjacent organs invasion (Number 4 patient, the right kidney were invaded; Number 10 patient, tumor invaded the descending segment of duodenum) and 2 cases occurred extrahepatic lymph node metastasis, as exhibited in Table 5. 4 cases presented with intrahepatic metastasis (the distance from the metastasis to the main lesion was more than 2 cm). Hepatic surface retraction was seen in 4 patients and no biliary dilatation periphery to tumor in all tumors. 3 cases were found ascites in CT.

**Table 5:** Quantitative CECT parameters of S-HCC patients

Patient number	Age/Gender	Attenuation of tumor major components (Hu)				Enhancement degree of CECT(Hu)			Dynamic enhancement pattern
		UP	AP	PVP	EP	EDA	EDP	EDE	
1	66/M	46	61	101	86	15	55	40	Slow-in and slow-out
2	59/M	41	60	82	75	19	41	34	Slow-in and slow-out
3	62/M	41	89	68	66	48	37	25	Wash-in and wash-out
4	45/M	45	68	98	80	23	53	35	Slow-in and slow-out
5	75/M	36	67	84	73	31	48	37	Wash-in and slow-out
6	56/F	40	48	62	59	8	22	19	Slow-in and slow-out
7	58/M	34	47	60	48	13	26	14	Slow-in and slow-out
8	59/M	49	63	83	73	14	34	24	Slow-in and slow-out
9	54/M	40	64	78	65	24	38	25	Slow-in and Slow-out
10	58/M	48	94	116	102	46	68	54	Wash-in and slow-out
11	50M	60	69	93	81	9	33	21	Slow-in and slow-out
12	81/F	35	110	117	126	75	82	91	Wash-in and slow-out
13	39/M	52	166	137	100	114	85	48	Wash-in and wash-out

*M* male, *F* female, *UP* unenhanced phase, *AP* arterial phase, *PVP* portal venous phase, *EP* equilibrium phase, *CECT* dynamic contrast enhanced CT

## Discussion

By investigating clinical characteristics and CECT features of 13 S-HCC patients, we found that S-HCCs were frequently seen in elderly male without any clinical symptoms but with HBV infection, elevated AFP and higher Ki-67 level. The CECT manifestations including: large diameter, frequently right lobe of liver involvement, obvious heterogeneity, a dominant enhancement pattern of “slow-in and and slow-out” and hepatic surface retraction were valuable clues to diagnose S-HCC. Moreover, lesions with lobular or wavy contours, ill-defined margins, infiltrative morphology and intrahepatic metastasis contributed to the diagnosis of S-HCC.

As a type of rare malignant tumor, S-HCC is usually characterized by atypical symptoms such as abdominal pain, weight loss, fatigue or discovered in routine medical examination without any symptoms, making diagnosis difficult at an early stage [15]. As our results showed that most patients complained with abdominal pain or tumors were found in asymptomatic physical examination, with a larger average diameter of 66.7mm. Moreover, most patients had HBV infection (53.8%, 7/13), but none of them had HCV infection, indicating that S-HCC was related to hepatitis B, to some extent. Several studies have revealed the similar phenomenon [6, 12, 15, 16]. As Lu et al. found that 19 patients (67.9 %) were positive for serum HBs antigen, and none were positive for HCV antibody in their study [16]. However, a recent meta-analysis demonstrated that there was no significant difference between S-HCC patients and conventional HCC patents in HBV infection [17]. These different results may be due to the small sample size, especially the lack of study in the population with HCV infection. A majority of previous studies found that AFP levels were increased in most S-HCC patients, while both this incidence and the degree of AFP elevation were lower compared to conventional HCC [2, 15, 18]. In our study, 53.8 % of patients had elevated AFP levels, similar to Wang’s and Li’s studies, suggesting that the elevation of AFP is not only an indication of conventional HCC, but also of S-HCC [15, 19].

Different from conventional HCC with a “wash-in and wash-out” enhancement pattern, most S-HCC in our study presented with the “slow-in and and slow-out” enhancement mode in CECT, which were similar to previous studies [7, 12, 13, 20, 21]. Since the major histopathological characteristics of S-HCC were the peripheral viable cancerous tissue accompanied by fibrous stroma, with central necrosis or hemorrhage [4, 7]. Peripheral tumor fibrous stroma may block or delay the flow of contrast agents in CECT examination, resulting in slow peripheral enhancement in the AP, progressive or persistent enhancement toward the tumor center in the PVP and mild descending enhancement in the EP, as our results displayed. In addition, owing to the sarcomatoid components with dominant part of poorly differentiated cells which were growing rapidly, the neovasculature was insufficient for blood and oxygen supplying, leading to central necrosis [7, 22]. As the ischemia or necrosis features in CECT were more frequently seen with a high frequency of 54% (7/13) in our cohort. Several studies reported that S-HCC with various enhancement patterns depended on the tissue component and it’s proportion, such as different proportion of hepatocellular, sarcomatous or necrosis components [5, 13]. The CT manifestations varied when certain histopathological components ranged from focal to prominent, leading to a dilemma of summarizing the typical image features of S-HCC. However, tumors with a “slow-in and and slow-out” enhancement pattern different from HCC should be taken S-HCC into account to some degree.

The imaging features of S-HCC were reported to be similar to those of ICC [21, 23]. For example, hepatic surface retraction, a typical feature closely associated with ICC, was seen in 4 patients (31%) in our study. A large amount of fibrous tissue accompanied by aggressive ICC cells would pull or invade adjacent subcapsular area, leading to hepatic surface retraction [24, 25]. We assumed that this similar feature may be attributed to the analogous pathological characteristics of the two malignant tumors, with peripheral viable cancerous tissue and fibrous stroma pulling adjacent liver tissue. Interestingly, there were three lesions located near the liver subcapsular area within the four cases, being easily to be shape of hepatic surface retraction, similar to Shi and his colleagues’ results [13]. In addition, among the four lesions with hepatic surface retraction, three tumors lacked capsules and one had partial capsule, indicating that most of them were prone to invade peripheral regions owing to the lack of limitation of complete capsule. Furthermore, it should be noted that the differential diagnosis should also include liver metastases, liver abscess, scirrhous subtype of HCC and primary hepatic sarcoma, which all could present as “slow-in and and slow-out” enhancement pattern, with larger size, lobulated or wavy contours and infiltrative morphology in some cases [12, 26, 27].

Most researches found that patients with S-HCC had higher extra-hepatic metastases, lymph node metastases and poor prognosis [5, 20, 21, 27]. Distinct genetic patterns of S-HCC compared to conventional HCC may contributed to their aggressive biological behaviors, in spite of the extreme lack of molecular studies about S-HCC. Zhang et al. demonstrated that S-HCC had a high rate of rearrangement and homozygous deletion in CDKN2A gene, a tumor suppressor gene causing a loss of gene funtion [6]. It was reported that genetic and epigenetic aberrations of CDKN2A resulted in enhanced carcinogenesis and poor prognosis in various cancers, such as ovarian cancer, lymphoma, and prostate cancer, etc [28, 29]. Hence, in agreement with previous studies, two patients with adjacent organs invasion (15.4%), two and four patients occurred lymph node metastasis (15.4%) and intrahepatic metastasis (30.7%), respectively, in our study, maybe partially owing to the CDKN2A mutation [5, 15, 17].

There were several limitations in our study should be noted. First, this was a single and small sample study as the very low prevalent rate of S-HCC, and since to it’s retrospective nature, selection bias were inevitable. Second, limited to most patients without MRI examinations in the early years as expensive costs, we didn’t analyze the MRI features of S-HCC. Third, we did not provide the prognosis and survival data of the patients, because most of them were lost in contact, leading to incomplete survival data of the entire study cohort. Finally, due to the absence of a control group in our study as limited patients sample, we were unable to generate statistical data on the difference between S-HCC and other hepatic tumors.

In conclusion, we explored and analyzed the CECT and clinical features of a series of S-HCC patients. S-HCC generally presented with obvious heterogeneity and a CTCT enhancing pattern of “slow-in and and slow-out”. The valuable clues in the diagnosis of S-HCC preoperatively also included: patients with HBV infection, elevated AFP level, large tumor size, irregular and unclear tumor margin, intrahepatic metastasis and hepatic surface retraction. Radiologists pay more attention to the above mentioned CT imaging findings and clinical data in detail might achieve an accurate diagnosis of S-HCC.
